# Socioeconomic and Eco-Environmental Drivers Differentially Trigger and Amplify Bacterial and Viral Outbreaks of Zoonotic Pathogens

**DOI:** 10.3390/microorganisms13030621

**Published:** 2025-03-07

**Authors:** Payton Phillips, Negin Nazari, Sneha Dharwadkar, Antoine Filion, Benedicta Essuon Akaribo, Patrick Stephens, Mekala Sundaram

**Affiliations:** 1 Center for Precision One Health and Department of Infectious Diseases, University of Georgia, Athens, GA 36688, USA; sneha.dharwadkar@uga.edu (S.D.); msundara@uga.edu (M.S.); 2 Savannah River Ecology Laboratory, University of Georgia, Aiken, SC 29808, USA; 3 Department of Integrative Biology, Oklahoma State University, Stillwater, OK 74078, USA; fnazari@okstate.edu (N.N.); afilion90@gmail.com (A.F.); benedicta.akaribo@okstate.edu (B.E.A.); patrick.stephens@okstate.edu (P.S.)

**Keywords:** infectious disease outbreak, outbreak drivers, zoonosis, virus, bacteria, socioeconomic factors, ecological and environmental factors, disease macroecology, transmission drivers

## Abstract

The frequency of infectious disease outbreaks and pandemics is rising, demanding an understanding of their drivers. Common wisdom suggests that increases in outbreak frequency are driven by socioeconomic factors such as globalization and urbanization, yet, the majority of disease outbreaks are caused by zoonotic pathogens that can be transmitted from animals to humans, suggesting the important role of ecological and environmental drivers. Previous studies of outbreak drivers have also failed to quantify the differences between major classes of pathogens, such as bacterial and viral pathogens. Here, we reconsider the observed drivers of a global sample of 300 zoonotic outbreaks, including the 100 largest outbreaks that occurred between 1977 and 2017. We show that socioeconomic factors more often trigger outbreaks of bacterial pathogens, whereas ecological and environmental factors trigger viral outbreaks. However, socioeconomic factors also act as amplifiers of viral outbreaks, with higher case numbers in viral outbreaks driven by a larger proportion of socioeconomic factors. Our results demonstrate that it is useful to consider the drivers of global disease patterns in aggregate due to commonalities that cross disease systems. However, our work also identifies important differences between the driver profiles of bacterial and viral diseases in aggregate.

## 1. Introduction

Understanding the spatial and temporal distribution of novel infectious disease events is among the most important tasks for the coming century [1,2,3]. According to the World Health Organization [4], nearly 10 million people die from infectious diseases each year. This includes a range of diseases such as respiratory infections, diarrheal diseases, tuberculosis, HIV/AIDS, malaria, and others. The overall frequency of disease outbreaks also appears to be increasing [2], and more than half of outbreaks and emerging infectious diseases (EID) events are zoonotic [1,2]. This indicates a critical need for a better understanding of the factors that cause and propagate disease outbreaks, particularly those caused by zoonotic pathogens that can be transmitted from animals to humans.

It has been speculated that increases in outbreak frequency are related to socioeconomic factors such as urbanization [5,6], agricultural activity [7], and globalization reviewed in [2,8,9]. However, the fact that the majority of disease events are caused by zoonotic pathogens [1,2] implies that environmental and ecological factors, including climate change [10], changes in the distribution and abundance of vectors [11], and biodiversity loss [12], might play an unexpectedly large role. It is currently difficult to assess whether socioeconomic factors or eco-environmental factors predominate, because there have been so few quantitative global studies of disease dynamics. To date, most quantitative studies have been focused on individual pathogens, e.g., [13,14,15,16,17,18,19], or in some cases, individual disease events, e.g., [20,21,22]. There have been relatively few studies characterizing the drivers of outbreaks in general, that is, of many different types of diseases considered in aggregate, but see [1,2,23,24]. Furthermore, no global studies that we are aware of have tested for differences in the drivers of outbreaks of major natural classes of pathogens. There is a great need for a better understanding of how the drivers of disease dynamics can both be shared and differ among various disease systems.

For example, Stephens et al. [24] quantified the drivers of a global sample of 300 zoonotic outbreaks. They showed that the driver profiles of the largest outbreaks, with thousands or more cases, differed significantly from those of randomly selected outbreaks, and also showed that large outbreaks were more often caused by viral than bacterial pathogens. However, even this study did not explicitly consider whether and how the drivers observed generally differed between viral and bacterial pathogens considered in aggregate. From a disease transmission and management perspective, these groups exhibit many important differences. For one, there are no broad-spectrum antivirals analogous to antibiotics in wide use, and so the evolution of antimicrobial resistance is not an important factor in viral outbreaks [25]. Viruses also tend to evolve more quickly than bacteria due to shorter generation times, with some viral lineages showing the highest rates of molecular evolution known across the tree of life [26,27]. Perhaps because of this, viruses are also more often able to infect multiple and/or distantly related host species than bacterial pathogens [28]. Somewhat surprisingly, whether these and other differences in the characteristics of viruses and bacteria translate into differences in the drivers commonly associated with outbreaks has never been systematically tested. For example, whether the socioeconomic factors that are thought to be driving increases in overall outbreak frequency [2,9] are more important compared to ecological and environmental factors, in generating or amplifying viral or bacterial outbreaks, has never been directly quantified.

Here, we reconsider the reported drivers of a global sample of 300 zoonotic disease outbreaks [24]. We focus on two main questions: (1) are there significant differences in the relative importance of socioeconomic versus ecological and environmental factors between viral and bacterial outbreaks, and (2) does the proportion of socioeconomic factors that contribute to an outbreak influence variation in realized case numbers, and if so, is this effect strongest in outbreaks of viral or bacterial pathogens? Though we consider these analyses primarily proof-of-concept, we regard them as a first step towards illuminating variation in the specific triggers and amplifiers shaping the dynamics of bacterial and viral outbreaks across the globe.

## 2. Materials and Methods

This study is based on the outbreak data of Stephens et al. [24], which consist of a sample of outbreaks from a database of approximately 4000 zoonotic outbreaks that occurred between 1974 and 2017. It includes the 100 largest outbreaks (in terms of realized case numbers) and 200 additional outbreaks sampled at random from the entire database. Here, the particular pathogens we define as “zoonotic” follow the recommendations of several zoonotic specialist groups including the CDC [29], UK Health Ministry [30], and Pan American Health Organization [31]. However, in general, we include pathogens in which wild animal reservoirs are at least a somewhat frequent source of infection. We reaggregated the data of Stephens et al. [24] to the country level, assigning outbreaks that spread to more than one country to multiple rows (one for each country). In the end, we had 106 “large” outbreaks (42 bacterial and 64 viral) and 210 randomly sampled “background” outbreaks (175 bacterial and 35 viral).

Stephens et al. [24] scored the reported drivers of outbreaks using a schema of 48 potential drivers, which was further described in Stephens et al. [32]. It consists of a binary rubric of 48 potential drivers (Table 1) based on factors discussed in reviews and syntheses of the literature on zoonotic outbreaks, e.g., [33,34,35,36]. The schema is designed to represent a variety of different kinds of drivers including ecological [37,38], environmental [7,39], and socioeconomic [40,41,42] factors. The drivers of outbreaks are scored based on factors reported both in peer-reviewed literature, as well as high quality gray literature written by specialists, including ProMED e-mails [43], Morbidity and Mortality Weekly Reports [29], and WHO reports, e.g., [44,45]. For each individual outbreak, a given driver was scored as either (0), not reported as contributing to an outbreak or (1), reported as contributing to an outbreak by at least one source, with a source noted.

**Figure 1:**
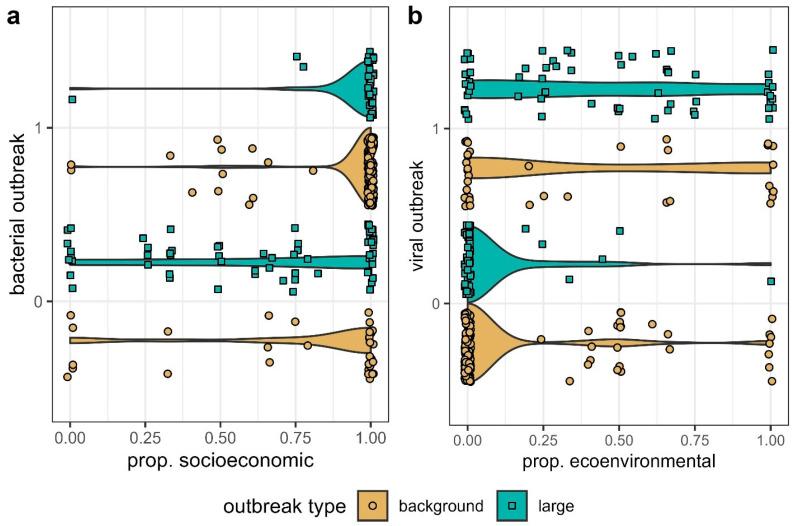
(**a**) Bacterial outbreak (1) vs. viral outbreak (0) based on the proportion of SE drivers for large and background outbreaks and (**b**) viral outbreak (1) vs. bacterial outbreak (0) based on the proportion of EE drivers for large and background outbreaks.

We divided the 48 drivers of Stephens et al. [24] into socioeconomic (SE) and/or ecological and environmental (EE) drivers (see Table 1). Socioeconomic drivers were those dealing primarily with social and economic aspects of human society, such as poverty, medical systems and interventions (for example antibiotics), cultural practices, trade, and travel. Ecological and environmental drivers were those pertaining to natural systems, such as weather, climate change, and changes to vector and reservoir populations, or interventions in natural systems such as the introduction and spread of invasive species. Some drivers were also considered both. For example, encroachment on wild areas occurs at the interface of settled and wild areas and is generally driven by SE factors. However, the disease transmission risk that it represents also largely depends on EE factors, such as the identity of wild animal hosts or vector abundance. The drivers we considered were in turn derived from synthetic reviews of the literature on zoonotic outbreaks (e.g., [33,34,36,46], see Stephens et al. (2021) for a more detailed description). For each outbreak, we calculated the proportion of drivers recorded as SE vs. EE (Figure 1).

### 2.1. Statistical Analyses

Using the data described above, we conducted two sets of analyses. Data and code for all analyses presented are included in the Supplementary Materials. All analyses were conducted in R v. 4.3.2 (Vienna, Austria) [47].

#### 2.1.1. Triggers of Bacterial vs. Viral Outbreaks

We tested whether bacterial vs. viral outbreaks tended to have a greater proportion of socioeconomic drivers or eco-environmental drivers. First, we used logistic regression to compare the type of pathogen that caused an outbreak, (0) viral or (1) bacterial, to the proportion of reported SE drivers (i.e., the proportion of drivers that were either SE alone or both). Then, we compared the type of outbreak, (1) viral or (0) bacterial, to the proportion of reported EE drivers (i.e., the proportion of outbreaks that were either EE alone or both). We included outbreak year as a covariate in both sets of models to account for possible differences in sampling effort, on the assumption that more recent outbreaks may be more thoroughly characterized than older outbreaks (see discussion of sample bias in Section 2.2 below). Finally, we conducted separate analyses of large and background outbreaks, for a total of four models. To ensure that the classification of drivers did not unduly influence our results, we conducted sensitivity analyses for our models with different definitions of EE and SE. First, we included the drivers classified as both SE and EE when calculating the proportion of either driver type. Second, we calculated the proportion of each driver type while excluding those categorized as both, essentially assuming that they were categorized as the opposing type of driver. We found that the classification of drivers did not influence our results and therefore report only the one set of analyses with “both” drivers in the main text (see Supplementary Materials Table S1 for analyses excluding “both” drivers).

#### 2.1.2. Amplifiers of Bacterial and Viral Outbreaks

Next, we considered whether the proportion of SE vs. EE drivers was related to variation in realized case numbers among outbreaks. Using negative binomial regression implemented in the R package MASS v. 7.3-61 [48], we compared the number of reported cases in each outbreak to the proportion of SE drivers, again including outbreak year as a covariate. We performed this analysis for (1) large bacterial outbreaks, (2) background bacterial outbreaks, (3) large viral outbreaks, and (4) background viral outbreaks. We parameterized each model twice, once with drivers categorized as both EE and SE drivers and once without. We found no qualitative difference in the models and report only the results with all drivers included here (see Supplementary Materials Table S2).

### 2.2. Sample Bias

Analyses of large spatiotemporal databases of diseases can introduce problems if reporting effort is not accounted for [2,24]. However, variation in sampling effort is unlikely to have affected our analyses. Our logistic regression analyses of viral vs. bacterial outbreaks would only be biased if there were some consistent difference in sampling effort between these two classes of pathogens. We are unaware of any evidence that suggests that bacterial or viral outbreaks tend to be consistently better studied. Stephens et al. [24] did show some evidence that large outbreaks were better studied, often being documented in more sources and having more reported drivers than background outbreaks. This is the reason that we conducted separate analyses of large and background outbreaks. Another possibility that has not, to our knowledge, been discussed previously, is that more recent outbreaks may tend to be better documented than older outbreaks, due to increases in the quality and quantity of communication infrastructure (e.g., phone and internet availability), medical technology, and other similar factors over time. In order to account for this possible bias, we also included outbreak start year as a covariate in all of our models. In addition, we explored additional potential sources of bias by including country-level gross domestic product (GDP) and population density in our models [49]. Past research has suggested that indicators of economic activity, such as GDP, may relate to improved health infrastructure and surveillance capabilities, leading to higher chances of outbreak detection [2,50]. More densely populated areas may also lead to higher outbreak detections [24]. The results of analyses that included the covariates were qualitatively identical with respect to the hypotheses tested. Furthermore, one of the models including these factors was overfitted and suffered from a lack of convergence. Therefore, we report only the results of models including year in the main text (for models including GDP and population density covariates, see Supplementary Materials Tables S3 and S4). Our results proved quite robust and generally consistent across all subsets of the data we considered, and similar regardless of whether bias covariates were significantly correlated with a given response variable (see results). This suggests that reporting efforts likely did not influence the recorded drivers in outbreaks and did not differentially influence viral vs. bacterial outbreak dynamics.

## 3. Results

We examined large and background outbreaks independently with logistic regressions. Across both datasets, our analyses found that bacterial outbreaks were positively related to the proportion of SE drivers recorded and not to outbreak start year (Table 2). The odds of a large outbreak being bacterial increased by e^6.35^ = 572 times when the proportion of SE drivers recorded increased from 0 to 1. Even for background outbreaks of smaller sizes, the odds of a bacterial outbreak increased by 17 times (e^2.83^) with a change from 0 to 100% SE drivers (Table 2). We observed similar patterns for the effect of EE drivers on viral outbreaks (Table 2). If the proportion of EE drivers changed from 0 to 1, the odds that an outbreak had viral origins increased by e^5.06^ = 158 times for large outbreaks and by e^2.45^ = 12 times for background outbreaks (Table 2).

**Figure 2:**
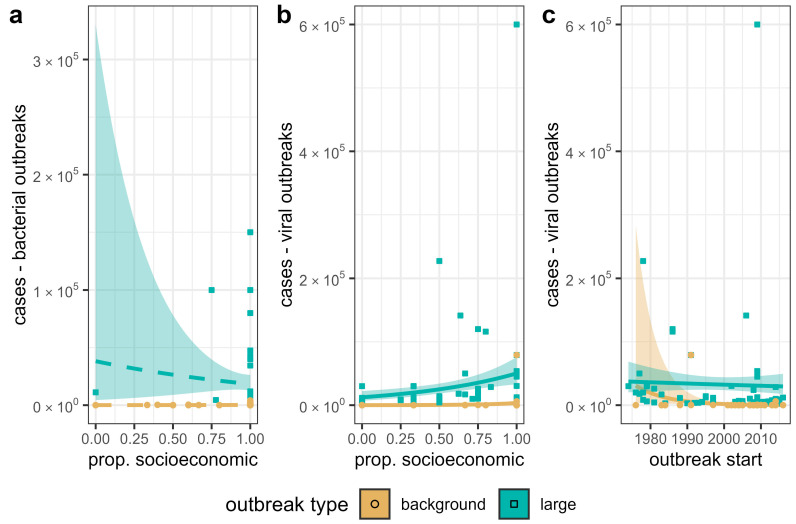
Results of negative binomial regressions predicting case numbers based on the proportion of SE drivers for (**a**) large and background bacterial outbreaks, (**b**) large and background viral outbreaks, and (**c**) based on year of outbreak start for viral outbreaks. Dashed lines indicate a non-significant relationship, while solid lines represent a significant relationship. Regressions are shown with 95% confidence intervals.

The size of bacterial pandemics and epidemics was not driven uniquely by SE or EE drivers. Our negative binomial regressions found no significant predictor of case numbers in either large or background bacterial outbreak datasets (Figure 2a; Table 3). Start year of the outbreak was also not related to case numbers (Table 3). Conversely, we found a positive slope for proportion of SE drivers and viral outbreak case numbers both in the large and background outbreak datasets (Figure 2b; Table 3). Case numbers increased by e^1.79^ = 6 times in large viral outbreaks, with an increase in the proportion of SE drivers from 0 to 1 (Table 3). For background viral outbreaks, case numbers increased 31 times (e^3.44^) when all SE drivers were reported, as opposed to no SE drivers reported (Table 3). Start year showed a small negative coefficient in predicting case numbers for viral outbreaks in both large and background outbreak datasets (Figure 2c; Table 3).

## 4. Discussion

Global increases in outbreak frequency [2] and disease emergence events [1] indicate a great need for more comprehensive management strategies that can potentially impact the dynamics of many different diseases. This is because it will be difficult to predict exactly where and when a particular disease may manifest next. However, infectious disease outbreaks are also caused by a wide variety of pathogens with varying characteristics. Furthermore, there is, as yet, no consensus as to whether socioeconomic (SE) factors such as globalization and urbanization or eco-environmental (EE) factors such as climate change, weather patterns, and the population dynamics of vectors are most important in driving global trends in outbreak frequency. As a proof-of-concept, we divided outbreaks along an obvious biological axis, considering whether the influence of SE vs. EE drivers differs between viral and bacterial outbreaks. The results of our analysis reveal important similarities and differences among outbreaks caused by different pathogens.

We show that SE factors were much more important in driving bacterial than viral outbreaks overall, with the chances that an outbreak was bacterial increasing drastically as the proportion of SE drivers that contributed to an outbreak increased (Table 2; Figure 1). These results corroborate previous works implicating socioeconomic triggers such as poverty, poor sanitation, and drug/antibiotic usage in the start of bacterial outbreaks [24,51,52]. For instance, some bacterial outbreaks result from the consumption of unwashed foods or contamination with fecal matter [24], while others begin from the evolution of drug resistance [53]. The latter factor has especially been cited as a growing threat to global public health [54].

In contrast to bacterial outbreaks, viral disease emergence is often driven by eco-environmental factors (Table 2; Figure 1), including climate change, changes in reservoir (or vector) abundances, and human–animal interface dynamics. These results are supported by separate statistical analyses for both large and background viral outbreaks (Table 2), as well as by past studies examining the origins of viral outbreaks [1,24,55]. For example, bat–human interfaces are known to initiate filoviral and henipaviral infections [32,56,57,58,59]. In general, resource use by bats is a key ecological driver of many bat-borne diseases [56,58,60]. Climatic conditions are additionally frequently cited as drivers of viral vector-borne diseases such as Crimean Congo Hemorrhagic Fever and Rift Valley fever [61,62].

Somewhat conversely, the proportion of SE factors rather than EE factors was important in amplifying viral outbreaks, with the expected case numbers increasing as the proportion of SE factors reported to drive an outbreak increased (Table 3, Figure 2b). This result supports the hypothesis that travel and trade, medical infrastructure, war, and other socioeconomic parameters increase case numbers and exacerbate the spread of viral pathogens [24,32,63,64,65]. The recent COVID-19 pandemic, which occurred after the most recent outbreaks included in the data we used for this study, is a prime example of a very large viral outbreak with case numbers driven by a myriad of socioeconomic drivers such as unequal access to healthcare, international/national travel, and vaccine hesitancy [65,66,67]. The rapid spread and rise in case numbers as a result of SE drivers mirrors the 6-fold increase in case numbers as SE drivers increased in our results.

In contrast to viral outbreaks, the proportion of EE vs. SE factors had little influence on case numbers reported in outbreaks of bacterial pathogens. Although the trends were in the same direction as those of viral outbreaks, they were not statistically significant in large or background bacterial outbreaks (Table 3, Figure 2a). This suggests that no single class of drivers is responsible for large epidemics of bacterial pathogens. Instead, bacterial outbreak size may be related to a synthesis of multiple socioeconomic and eco-environmental drivers or to a few specific drivers, rather than an aggregate of driver types. Stephens et al. [24] did examine what drivers are responsible for large outbreaks. Their conclusion was that specific critical failures, such as the contamination of a sewage system [68] or large-scale food contamination events [69], which would both be categorized as socioeconomic drivers in our analysis, typically resulted in a greater spread of bacteria.

Just as important as the differences that we found between viral and bacterial outbreaks was the fact that within each class of pathogen, the results were surprisingly consistent across large and background outbreaks. Though the strength of the relationships observed varied somewhat, the qualitative results were highly consistent. The proportion of EE drivers influenced the chances that both large and background outbreaks were caused by a viral pathogen. Perhaps more importantly, the fact that viral outbreaks with more SE drivers tended to be larger, with more reported cases, was also consistent between large and background viral outbreaks. This is in sharp contrast to the results of Stephens et al. [24], which showed significant differences in the driver profile of large vs. background outbreaks. The consistency of our results likely indicates that even taking simple biological differences between pathogens, viral vs. bacterial in the case of our analyses, can help make commonalities among outbreaks of different diseases more apparent. We also speculate that aggregating all drivers into one of two broad categories (i.e., SE or EE), instead of using the more granular approach of Stephens et al. [24,32], may have also helped reveal commonalities between large and background outbreaks.

For the first time, we also considered the potential of the year in which an outbreak occurred to bias our results. We did this by including outbreak start year as a covariate in all of our analyses. Smith et al. [2] showed that factors such as number of phone lines and number of internet users, which in most countries have tended to increase over time [49], can bias disease data, with more outbreaks generally documented in countries with better communication infrastructure. Improvements in medical technology and advances in epidemiology as a discipline over time could also change how disease outbreaks are understood and investigated. However, we found no evidence that these factors biased our analyses. Generally, there was no relationship between outbreak start year and any response variable we considered. We did find a small and negative effect of start year on case numbers for viral outbreaks (Table 3). However, we suggest that this effect is reflective of the raw data that was published prior to the COVID-19 pandemic (see original publication and data in Stephens et al. [24]). We speculate that if more recent outbreaks such as Monkeypox (N = 102,000 cases [70]) and COVID-19 (N = 777,315,739 cases [71]) were included, this parameter would not be significant.

Another potential source of bias that we did not directly consider is that our scored drivers reflect only the most discussed drivers, which may not always include the true predictors of infectious diseases. Certainly, the drivers examined in this study were gathered from published narratives describing outbreaks (see Stephens et al. [24] for details), which might be subject to biases. However, scored drivers do statistically differentiate large outbreaks, as shown in Stephens et al. [24], and filovirus outbreaks, as explored in Stephens et al. [32]. These unique reported drivers associated with many different types of outbreaks could provide important indicators of infectious disease spread that warrant more attention. Quantifying the critical socioeconomic drivers triggering bacterial outbreaks and/or leading to the spread of viral cases is an important next step. Similarly, quantifying the EE triggers of viral disease outbreaks is necessary in order to forecast when and where new outbreaks will arise. In doing so, we may be able to prevent outbreaks or address them early on, before case numbers rise due to other factors.

## 5. Conclusions

Overall, we suggest that local conditions can enhance the chances of specific types of outbreaks [72]. Although this finding is not new, very few have investigated broad drivers and their association with broad pathogen classes. We propose that this angle of investigation opens up new areas of insights. For example, if broader groups of pathogens, such as bacteria and viruses, demonstrate unique mechanistic triggers and drivers of case numbers, then other broad classes of pathogens, such as protozoa and helminths, or those sharing similar transmission modes, are also likely to show important commonalities and differences [72]. Our work points to a great need for more research to quantify and classify the factors that contribute to major classes of infectious disease outbreaks.

## Figures and Tables

**Table 1:** Schema of 48 potential drivers used to score outbreaks. Drivers are considered socioeconomic (SE), eco-environmental (EE), or both (B). Drivers are ordered based on the total number of outbreaks in which they were noted as a driver.

Driver	Type	Count
Food contamination	SE	118
Water contamination	SE	82
Local livestock production	SE	54
Sewage management (poor management practices, inadequate infrastructure, or structural failures)	SE	51
Weather conditions	EE	47
International travel/trade	SE	43
Change in vector abundance	EE	33
Human–animal contact (not including vectors)	B	33
Public health infrastructure (inadequate infrastructure, equipment, personnel, or monitoring)	SE	29
Antibiotics (e.g., antibiotic-resistant strains)	SE	22
Medical procedures (misdiagnosis, incorrect procedures, or failure of correct procedures)	SE	21
Industrial livestock production	SE	19
Vaccination breakdown (failure to administer)	SE	18
Human population density	SE	17
Livestock and domestic–wildlife interface	B	16
War/conflict	SE	15
Poverty (miscellaneous stressors related to poverty and/or marginalization)	SE	14
Intranational travel/trade	SE	13
Natural disasters	EE	13
Change in reservoir abundance	EE	12
Agricultural activity	SE	11
Climate change (change in long-term trends)	EE	8
Wetland cultivation	SE	6
Soil contamination	SE	6
Cultural/religious beliefs or practices (which conflict with best health and medical practices)	SE	6
Deforestation	SE	5
Socioeconomic change (e.g., broad-scale changes in governance)	SE	5
Change in vector distribution	EE	4
Encroachment	B	4
Wildlife hunting (including capture, processing, consumption; often referred to as “bushmeat”)	SE	4
Change in reservoir distribution	EE	3
Vector control (change in or reportedly deficient vector control practices)	EE	3
Immunosuppression	SE	3
Malnourishment (e.g., nutrient deficiency or lack of food in particular social groups)	SE	3
Introduced/invasive species (free living species including vectors, but not invasive pathogens)	EE	2
Mining	SE	2
Dam building	SE	2
Co-infection	SE	2
Wildlife provisioning	SE	1
Logging	SE	1
Human demographic change	SE	1
Road building	SE	1
Ineffective vaccine	SE	1
Famine (widespread nearly universal shortage of food in a region)	SE	1
Aquaculture	SE	0
Irrigation	SE	0
Reforestation	SE	0
Urbanization	SE	0

**Table 2:** Summary of logistic regressions predicting outbreak type (bacterial [1] vs. viral [0] or viral [1] vs. bacterial [0]) for large and background outbreaks. For bacterial outbreaks, the proportion of socioeconomic (SE) drivers reported in the literature was used as a predictor, while the proportion of eco-environmental (EE) drivers reported in the literature was used as a predictor for viral outbreaks. Start year of the outbreak was used as a predictor in all models. This table summarizes coefficient estimates, standard error around estimate, z values, and accompanying *p*-values. Bold-faced rows indicate statistically significant drivers (*p* < 0.05).

Outbreak Type	Dataset	Variable	Est.	SE	*z* Value	Pr(>|x|)
Bacterial (1) vs. viral (0)	Large	Intercept	65.4	40.1	1.63	0.103
		**Prop. SE drivers**	**6.35**	**1.72**	**3.68**	**<0.001**
		Start year	−0.036	0.02	−1.76	0.079
	Background	Intercept	44.5	47.7	0.932	0.352
		**Prop. SE drivers**	**2.83**	**0.720**	**3.93**	**<0.001**
		Start year	−0.023	0.024	−0.950	0.342
Viral (1) vs. bacterial (0)	Large	Intercept	−57.5	39.4	−1.46	0.144
		**Prop. EE drivers**	**5.06**	**1.19**	**4.25**	**<0.001**
		Start year	0.029	0.020	1.45	0.148
	Background	Intercept	−53.1	47.9	−1.11	0.268
		**Prop. EE drivers**	**2.45**	**0.534**	**4.59**	**<0.001**
		Start year	0.025	0.024	1.07	0.287

**Table 3:** Summary of negative binomial regressions predicting case numbers of large and background bacterial and viral outbreaks. For all regressions, the proportion of SE drivers reported in the literature and start year of outbreak were used as predictors. This table summarizes coefficient estimates, standard error around estimate, z values, and accompanying *p*-values. Bold-faced rows indicate statistically significant drivers (*p* < 0.05).

Outbreak Type	Dataset	Variable	Est.	SE	*z* Value	Pr(>|x|)
Bacterial	Large	Intercept	33.4	33.7	0.992	0.321
		Prop. SE drivers	−1.11	1.15	−0.966	0.334
		Start year	−0.011	0.017	−0.673	0.501
	Background	Intercept	19.4	26.1	0.743	0.458
		Prop. SE drivers	1.11	0.717	1.55	0.122
		Start year	−0.008	0.013	−0.587	0.557
Viral	Large	**Intercept**	**56.5**	**21.4**	**2.64**	**0.008**
		**Prop. SE drivers**	**1.79**	**0.397**	**4.49**	**<0.001**
		**Start year**	**−0.024**	**0.011**	**−2.21**	**0.027**
	Background	**Intercept**	**233**	**73.4**	**3.18**	**0.001**
		**Prop. SE drivers**	**3.44**	**0.947**	**3.63**	**<0.001**
		**Start year**	**−0.115**	**0.037**	**−3.12**	**0.002**

## Data Availability

The data and analyses presented in this study are included in the Supplementary Materials. Original data used are available in the Supplementary Materials of Stephens et al., 2021 [24].

## Supplementary Materials

The following supporting information can be downloaded at: https://www.mdpi.com/article/10.3390/microorganisms13030621/s1, Table S1: Summary of logistic regressions predicting outbreak type for large and background outbreaks with alternate parameterization of driver type; Table S2: Summary of alternate negative binomial regressions predicting case numbers of large and background bacterial and viral outbreaks with alternate parameterization of driver type; Table S3: Summary of logistic regressions predicting outbreak type for large and background outbreaks with additional variables; Table S4: Summary of negative binomial regressions predicting case numbers of large and background bacterial and viral outbreaks with additional variables; background_outbreaks_v2: Raw data file of drivers for 210 randomly sampled bacterial and viral “background” outbreaks; large_outbreaks_v2: Raw data file of drivers for 106 largest bacterial and viral outbreaks, R code: R code utilizing the data in background_outbreaks_v2 and large_outbreaks_v2 to run all analyses included in this paper.

## Abbreviations

The following abbreviations are used in this manuscript:

SE	Socioeconomic
EE	Ecological and environmental

## References

[1] Jones K.E., Patel N.G., Levy M.A., Storeygard A., Balk D., Gittleman J.L., Daszak P. (2008). Global Trends in Emerging Infectious Diseases. Nature.

[2] Smith K.F., Goldberg M., Rosenthal S., Carlson L., Chen J., Chen C., Ramachandran S. (2014). Global Rise in Human Infectious Disease Outbreaks. J. R. Soc. Interface.

[3] Khorram-Manesh A., Goniewicz K., Burkle F.M. (2024). Unleashing the Global Potential of Public Health: A Framework for Future Pandemic Response. J. Infect. Public Health.

[4] World Health Organization (WHO) The Top 10 Causes of Death. https://www.who.int/news-room/fact-sheets/detail/the-top-10-causes-of-death.

[5] Clegg E.J., Garlick J.P. (2022). Disease and Urbanization.

[6] Neiderud C.-J. (2015). How Urbanization Affects the Epidemiology of Emerging Infectious Diseases. Infect. Ecol. Epidemiol..

[7] Jones B.A., Grace D., Kock R., Alonso S., Rushton J., Said M.Y., McKeever D., Mutua F., Young J., McDermott J. (2013). Zoonosis Emergence Linked to Agricultural Intensification and Environmental Change. Proc. Natl. Acad. Sci. USA.

[8] Smith K.F., Sax D.F., Gaines S.D., Guernier V., Guégan J.-F. (2007). Globalization of Human Infectious Disease. Ecology.

[9] Sigler T., Mahmuda S., Kimpton A., Loginova J., Wohland P., Charles-Edwards E., Corcoran J. (2021). The Socio-Spatial Determinants of COVID-19 Diffusion: The Impact of Globalisation, Settlement Characteristics and Population. Glob. Health.

[10] Lafferty K.D. (2009). The Ecology of Climate Change and Infectious Diseases. Ecology.

[11] Chaves L.F., Hamer G.L., Walker E.D., Brown W.M., Ruiz M.O., Kitron U.D. (2011). Climatic Variability and Landscape Heterogeneity Impact Urban Mosquito Diversity and Vector Abundance and Infection. Ecosphere.

[12] Wilkinson D.A., Marshall J.C., French N.P., Hayman D.T.S. (2018). Habitat Fragmentation, Biodiversity Loss and the Risk of Novel Infectious Disease Emergence. J. R. Soc. Interface.

[13] Deyle E.R., Maher M.C., Hernandez R.D., Basu S., Sugihara G. (2016). Global Environmental Drivers of Influenza. Proc. Natl. Acad. Sci. USA.

[14] Schmidt J.P., Park A.W., Kramer A.M., Han B.A., Alexander L.W., Drake J.M. (2017). Spatiotemporal Fluctuations and Triggers of Ebola Virus Spillover. Emerg. Infect. Dis..

[15] da Costa A.C.C., Codeço C.T., Krainski E.T., Gomes M.F.d.C., Nobre A.A. (2018). Spatiotemporal Diffusion of Influenza A (H1N1): Starting Point and Risk Factors. PLoS ONE.

[16] Dewey-Mattia D. (2018). Surveillance for Foodborne Disease Outbreaks—United States, 2009–2015. MMWR Surveill. Summ..

[17] Siraj A.S., Rodriguez-Barraquer I., Barker C.M., Tejedor-Garavito N., Harding D., Lorton C., Lukacevic D., Oates G., Espana G., Kraemer M.U.G. (2018). Data Descriptor: Spatiotemporal Incidence of Zika and Associated Environmental Drivers for the 2015–2016 Epidemic in Colombia. Sci. Data.

[18] Laneri K., Cabella B., Prado P.I., Coutinho R.M., Kraenkel R.A. (2019). Climate Drivers of Malaria at Its Southern Fringe in the Americas. PLoS ONE.

[19] Wu T. (2021). The Socioeconomic and Environmental Drivers of the COVID-19 Pandemic: A Review. Ambio.

[20] Medaglia M.L.G., Pereira A.d.C., Freitas T.R.P., Damaso C.R. (2011). Swinepox Virus Outbreak, Brazil, 2011. Emerg. Infect. Dis..

[21] Goldani L.Z. (2018). Measles Outbreak in Brazil, 2018. Braz. J. Infect. Dis..

[22] Kock R.A., Begovoeva M., Ansumana R., Suluku R. (2019). Searching for the source of Ebola: The elusive factors driving its spillover into humans during the West African outbreak of 2013–2016. Rev. Sci. Tech..

[23] Semenza J.C., Lindgren E., Balkanyi L., Espinosa L., Almqvist M.S., Penttinen P., Rocklöv J. (2016). Determinants and Drivers of Infectious Disease Threat Events in Europe. Emerg. Infect. Dis..

[24] Stephens P.R., Gottdenker N., Schatz A.M., Schmidt J.P., Drake J.M. (2021). Characteristics of the 100 Largest Modern Zoonotic Disease Outbreaks. Phil. Trans. R. Soc. B.

[25] Hwang A.Y., Gums J.G. (2016). The Emergence and Evolution of Antimicrobial Resistance: Impact on a Global Scale. Bioorganic Med. Chem..

[26] Duffy S., Shackelton L.A., Holmes E.C. (2008). Rates of Evolutionary Change in Viruses: Patterns and Determinants. Nat. Rev. Genet..

[27] Peck K.M., Lauring A.S. (2018). Complexities of Viral Mutation Rates. J. Virol..

[28] Shaw L.P., Wang A.D., Dylus D., Meier M., Pogacnik G., Dessimoz C., Balloux F. (2020). The Phylogenetic Range of Bacterial and Viral Pathogens of Vertebrates. Mol. Ecol..

[29] CDC Completed OHZDP Workshops. https://www.cdc.gov/one-health/php/prioritization/completed-workshops.html?CDC_AAref_Val=https://www.cdc.gov/onehealth/what-we-do/zoonotic-disease-prioritization/completed-workshops.html.

[30] UK Public Health England List of Zoonotic Diseases. https://www.gov.uk/government/publications/list-of-zoonotic-diseases/list-of-zoonotic-diseases.

[31] Acha P.N., Szyfres B. (2005). Zoonoses and Communicable Diseases Common to Man and Animals.

[32] Stephens P.R., Sundaram M., Ferreira S., Gottdenker N., Nipa K.F., Schatz A.M., Schmidt J.P., Drake J.M. (2022). Drivers of African Filovirus (Ebola and Marburg) Outbreaks. Vector Borne Zoonotic Dis..

[33] Lederberg J., Shope R.E., Oaks S.C., Institute of Medicine (1993). Emerging Infections: Microbial Threats to Health in the United States.

[34] Smolinski M.S., Hamburg M.A., Lederberg J., Institute Of Medicine (2003). Microbial Threats to Health: Emergence, Detection, and Response.

[35] Ceddia M.G., Bardsley N.O., Goodwin R., Holloway G.J., Nocella G., Stasi A. (2013). A Complex System Perspective on the Emergence and Spread of Infectious Diseases: Integrating Economic and Ecological Aspects. Ecol. Econ..

[36] Gottdenker N.L., Streicker D.G., Faust C.L., Carroll C.R. (2014). Anthropogenic Land Use Change and Infectious Diseases: A Review of the Evidence. EcoHealth.

[37] Patz J.A., Daszak P., Tabor G.M., Aguirre A.A., Pearl M., Epstein J., Wolfe N.D., Kilpatrick A.M., Foufopoulos J., Molyneux D. (2004). Unhealthy Landscapes: Policy Recommendations on Land Use Change and Infectious Disease Emergence. Environ. Health Perspect..

[38] Schmeller D.S., Courchamp F., Killeen G. (2020). Biodiversity Loss, Emerging Pathogens and Human Health Risks. Biodivers. Conserv..

[39] Daszak P., Cunningham A.A., Hyatt A.D. (2001). Anthropogenic Environmental Change and the Emergence of Infectious Diseases in Wildlife. Acta Trop..

[40] Molyneux D., Hallaj Z., Keusch G.T., McManus D.P., Ngowi H., Cleaveland S., Ramos-Jimenez P., Gotuzzo E., Kar K., Sanchez A. (2011). Zoonoses and Marginalised Infectious Diseases of Poverty: Where Do We Stand?. Parasites Vectors.

[41] Grace D., Mutua F., Ochungo P., Kruska R., Jones K., Brierley L., Lapar L., Said M., Herrero M., Duc Phuc P. (2012). Mapping of Poverty and Likely Zoonoses Hotspots.

[42] Wu T., Perrings C., Kinzig A., Collins J.P., Minteer B.A., Daszak P. (2017). Economic Growth, Urbanization, Globalization, and the Risks of Emerging Infectious Diseases in China: A Review. Ambio.

[43] Madoff L.C. (2004). ProMED-Mail: An Early Warning System for Emerging Diseases. Clin. Infect. Dis..

[44] WHO (1978). Ebola Haemorrhagic Fever in Sudan, 1976. Bull. World Health Organ..

[45] WHO (2005). Outbreak of Ebola Haemorrhagic Fever in Yambio, South Sudan, April–June 2004. Wkly. Epidemiol. Rec..

[46] Kilpatrick A.M., Randolph S.E. (2012). Drivers, Dynamics, and Control of Emerging Vector-Borne Zoonotic Diseases. Lancet.

[47] R Core Team (2024). R: A Language and Environment for Statistical Computing.

[48] Venables W.N., Ripley B.D., Chambers J., Eddy W., Hardle W., Sheather S., Tierney L. (2002). Modern Applied Statistics with S.

[49] World Bank Open Data. https://data.worldbank.org/.

[50] Kluberg S.A., Mekaru S.R., McIver D.J., Madoff L.C., Crawley A.W., Smolinski M.S., Brownstein J.S. (2016). Global Capacity for Emerging Infectious Disease Detection, 1996–2014. Emerg. Infect. Dis..

[51] Irfan M., Almotiri A., AlZeyadi Z.A. (2022). Antimicrobial Resistance and Its Drivers—A Review. Antibiotics.

[52] Chang H.-H., Cohen T., Grad Y.H., Hanage W.P., O’Brien T.F., Lipsitch M. (2015). Origin and Proliferation of Multiple-Drug Resistance in Bacterial Pathogens. Microbiol. Mol. Biol. Rev..

[53] Davies J., Davies D. (2010). Origins and Evolution of Antibiotic Resistance. Microbiol. Mol. Biol. Rev..

[54] Murray C.J.L., Ikuta K.S., Sharara F., Swetschinski L., Aguilar G.R., Gray A., Han C., Bisignano C., Rao P., Wool E. (2022). Global Burden of Bacterial Antimicrobial Resistance in 2019: A Systematic Analysis. Lancet.

[55] Allen T., Murray K.A., Zambrana-Torrelio C., Morse S.S., Rondinini C., Di Marco M., Breit N., Olival K.J., Daszak P. (2017). Global Hotspots and Correlates of Emerging Zoonotic Diseases. Nat. Commun..

[56] Kuhn J.H. (2008). Filoviruses. A Compendium of 40 Years of Epidemiological, Clinical, and Laboratory Studies. Arch. Virol. Suppl..

[57] Plowright R.K., Eby P., Hudson P.J., Smith I.L., Westcott D., Bryden W.L., Middleton D., Reid P.A., McFarlane R.A., Martin G. (2015). Ecological Dynamics of Emerging Bat Virus Spillover. Proc. Biol. Sci..

[58] Eby P., Peel A.J., Hoegh A., Madden W., Giles J.R., Hudson P.J., Plowright R.K. (2023). Pathogen Spillover Driven by Rapid Changes in Bat Ecology. Nature.

[59] Olival K.J., Islam A., Yu M., Anthony S.J., Epstein J.H., Khan S.A., Khan S.U., Crameri G., Wang L.-F., Lipkin W.I. (2013). Ebola Virus Antibodies in Fruit Bats, Bangladesh. Emerg. Infect. Dis..

[60] Filion A., Sundaram M., Stephens P.R. (2023). Preliminary Investigation of Schmalhausen’s Law in a Directly Transmitted Pathogen Outbreak System. Viruses.

[61] Jameson L.J., Ramadani N., Medlock J.M. (2012). Possible Drivers of Crimean-Congo Hemorrhagic Fever Virus Transmission in Kosova. Vector Borne Zoonotic Dis..

[62] Lancelot R., Béral M., Rakotoharinome V.M., Andriamandimby S.-F., Héraud J.-M., Coste C., Apolloni A., Squarzoni-Diaw C., De La Rocque S., Formenty P.B.H. (2017). Drivers of Rift Valley Fever Epidemics in Madagascar. Proc. Natl. Acad. Sci. USA.

[63] Olivero J., Guilbert E. (2021). Biogeography of Diseases. Biogeography: An Integrative Approach of the Evolution of Living.

[64] Sundaram M., Filion A., Akaribo B.E., Stephens P.R. (2023). Footprint of War: Integrating Armed Conflicts in Disease Ecology. Trends Parasitol..

[65] Núñez A., Sreeganga S.D., Ramaprasad A. (2021). Access to Healthcare during COVID-19. Int. J. Environ. Res. Public Health.

[66] Morens D.M., Daszak P., Markel H., Taubenberger J.K. (2020). Pandemic COVID-19 Joins History’s Pandemic Legion. mBio.

[67] Huang C., Yang L., Pan J., Xu X., Peng R. (2022). Correlation between Vaccine Coverage and the COVID-19 Pandemic throughout the World: Based on Real-world Data. J. Med. Virol..

[68] Kauppinen A., Pitkänen T., Al-Hello H., Maunula L., Hokajärvi A.-M., Rimhanen-Finne R., Miettinen I.T. (2019). Two Drinking Water Outbreaks Caused by Wastewater Intrusion Including Sapovirus in Finland. Int. J. Environ. Res. Public Health.

[69] Tauxe R.V. (1997). Emerging Foodborne Diseases: An Evolving Public Health Challenge. Emerg. Infect. Dis..

[70] CDC Ongoing Clade II Mpox Global Outbreak. https://www.cdc.gov/mpox/outbreaks/2022/index-1.html.

[71] WHO WHO COVID-19 Dashboard. https://data.who.int/dashboards/covid19/cases.

[72] Eisenberg J.N.S., Desai M.A., Levy K., Bates S.J., Liang S., Naumoff K., Scott J.C. (2007). Environmental Determinants of Infectious Disease: A Framework for Tracking Causal Links and Guiding Public Health Research. Environ. Health Perspect..

