# Supplementary material for: Socioeconomic and Eco-Environmental Drivers Differentially Trigger and Amplify Bacterial and Viral Outbreaks of Zoonotic Pathogens
Source: Microorganisms. 2025 Mar 7;13(3):621. doi: 10.3390/microorganisms13030621 (PMC11945676; doi:10.3390/microorganisms13030621)
Supplement: Supplementary file 1 [file microorganisms-13-00621-s001.zip › Supplementary Materials.pdf]

Table S1. Summary of logistic regressions predicting outbreak type (bacterial [1] vs viral [0] or viral [1] vs. bacterial [0]) for large and background outbreaks. For bacterial outbreaks, the proportion of socioeconomic (SE) drivers reported in literature was used as a predictor, while the proportion of eco-environmental (EE) drivers reported in literature was used as a predictor for viral outbreaks. In this alternate analysis, we included only those drivers categorized as SE or EE and not both. Start year of the outbreak was used as a predictor in all models. This table summarizes coefficient estimates, standard error around estimate, z values and accompanying p-values. Bold-faced rows indicate statistically significant drivers (p<0.05).

Outbreak Type	Dataset	Variable	Est.	SE	z value	Pr(>|x|)
Bacterial (1) vs viral (0)	Large	Intercept	52.4	39.2	1.34	0.181
		**Prop. *SE* drivers**	**5.06**	**1.19**	**4.25**	**<0.001**
		Start Year	-0.029	0.020	**-1.45**	0.148
	Background	Intercept	50.6	47.8	1.06	0.290
		**Prop. *SE* drivers**	**2.45**	**0.534**	**4.59**	**<0.001**
		Start Year	-0.025	0.024	-1.07	0.287
Viral (1) vs. bacterial (0)	Large	Intercept	-71.7	40.6	-1.77	0.077
		**Prop. *EE* drivers**	**6.35**	**1.72**	**3.68**	**<0.001**
		Start year	0.036	0.020	1.76	0.079
	Background	Intercept	-47.3	47.8	-0.989	0.323
		**Prop. *EE* drivers**	**2.83**	**0.720**	**3.93**	**<0.001**
		Start year	0.023	0.024	0.950	0.342

Table S2. Summary of negative binomial regressions predicting case numbers of large and background bacterial and viral outbreaks. For all regressions, the proportion of SE drivers reported in literature and start year of outbreak were used as predictors. In this alternate analysis, we included only those drivers categorized as SE and not both. This table summarizes coefficient estimates, standard error around estimate, z values and accompanying p-values. Bold-faced rows indicate statistically significant drivers (p<0.05).

Outbreak Type	Dataset	Variable	Est.	SE	z value	Pr(>|x|)
Bacterial	Large	Intercept	19.6	33.9	0.578	0.563
		Prop. *SE* drivers	0.252	0.976	0.259	0.796
		Start Year	-0.005	0.017	-0.296	0.767
	Background	Intercept	34.3	26.3	1.31	0.191
		Prop. *SE* drivers	-0.406	0.459	-0.884	0.377
		Start Year	-0.014	0.013	-1.10	0.274
Viral	Large	Intercept	32.9	22.6	1.46	0.145
		**Prop. *SE* drivers**	**1.04**	**0.428**	**2.43**	**0.015**
		Start year	-0.011	0.011	-1.02	0.306
	Background	**Intercept**	**222**	**67.3**	**3.31**	**<0.001**
		**Prop. *SE* drivers**	**3.93**	**0.738**	**5.33**	**<0.001**
		**Start year**	**-0.109**	**0.034**	**-3.26**	**0.001**

Table S3. Summary of logistic regressions predicting outbreak type (bacterial [1] vs viral [0] or viral [1] vs. bacterial [0]) for large and background outbreaks. For bacterial outbreaks, the proportion of socioeconomic (SE) drivers reported in literature was used as a predictor, while the proportion of eco-environmental (EE) drivers reported in literature was used as a predictor for viral outbreaks. Start year of the outbreak was used as a predictor in all models. In this alternative analysis, we also included country-level GDP and population in the year of the outbreak as predictors. This table summarizes coefficient estimates, standard error around estimate, z values and accompanying p-values. Bold-faced rows indicate statistically significant drivers (p<0.05).

Outbreak Type	Dataset	Variable	Est.	SE	z value	Pr(>|x|)
Bacterial (1) vs viral (0)	Large	Intercept	53.9	42.1	1.28	0.200
		**Prop. *SE* drivers**	6.55	1.82	**3.60**	**<0.001**
		Start Year	-0.03	0.021	-1.41	0.158
		Log(gdp)	-0.467	0.271	-1.73	0.085
		Log(population)	-0.337	0.279	-1.21	0.227
	Background	Intercept	61.8	53.0	1.17	0.244
		**Prop. *SE* drivers**	**3.23**	**0.814**	**3.97**	**<0.001**
		Start Year	-0.031	0.027	-1.19	0.236
		**Log(gdp)**	**0.597**	**0.203**	**2.94**	**0.003**
		Log(population)	-0.170	0.209	-0.813	0.416
Viral (1) vs. bacterial (0)	Large	Intercept	-47.7	41.4	-1.15	0.250
		**Prop. *EE* drivers**	**5.17**	**1.28**	**4.03**	**<0.001**
		Start year	0.024	0.021	1.14	0.255
		Log(gdp)	0.366	0.266	1.38	0.169
		Log(population)	0.415	0.283	1.47	0.142
	Background	Intercept	-74.5	54.0	-1.38	0.168
		**Prop. *EE* drivers**	**3.20**	**0.648**	**4.94**	**<0.001**
		Start year	0.036	0.027	1.33	0.182
		**Log(gdp)**	**-0.708**	**0.218**	**-3.25**	**0.001**
		Log(population)	0.260	0.225	1.16	0.247

Table S4. Summary of negative binomial regressions predicting case numbers of large and background bacterial and viral outbreaks. For all regressions, the proportion of SE drivers reported in literature and start year of outbreak were used as predictors. In this alternative analysis, we also included country-level GDP and population in the year of the outbreak as predictors. Note that the full model for background viral outbreaks did not converge. This table summarizes coefficient estimates, standard error around estimate, z values and accompanying p-values. Bold-faced rows indicate statistically significant drivers ($p<0.05$).

Outbreak Type	Dataset	Variable	Est.	SE	z value	Pr(>|x|)
Bacterial	Large	Intercept	26.2	34.0	0.772	0.440
		Prop. *SE* drivers	-0.801	1.16	-0.689	0.491
		Start Year	-0.008	0.017	-0.463	0.644
		Log(gdp)	0.120	0.183	0.654	0.513
		Log(population)	-0.053	0.230	-0.230	0.818
	Background	Intercept	22.1	26.0	0.850	0.395
		Prop. *SE* drivers	1.00	0.713	1.41	0.159
		Start Year	-0.009	0.013	-0.692	0.489
		**Log(gdp)**	**-0.306**	**0.113**	**-2.71**	**0.007**
		**Log(population)**	**0.439**	**0.108**	**4.08**	**<0.001**
Viral	Large	**Intercept**	**78.1**	**24.1**	**3.24**	**0.001**
		**Prop. S*E* drivers**	**0.962**	**0.353**	**2.72**	**0.006**
		**Start year**	**-0.034**	**0.012**	**-2.85**	**0.004**
		Log(gdp)	0.128	0.174	0.735	0.462
		Log(population)	-0.253	0.135	-1.88	0.060
	Background	**Intercept**	**6.45**	**0.349**	**18.5**	**<0.001**
		**Log(gdp)**	**0.708**	**0.307**	**2.31**	**0.021**
		**Log(population)**	**2.21**	**0.349**	**6.35**	**<0.001**
